# The influence of ACE ID and ACTN3 R577X polymorphisms on lower-extremity function in older women in response to high-speed power training

**DOI:** 10.1186/1471-2318-13-131

**Published:** 2013-12-06

**Authors:** Ana Pereira, Aldo M Costa, José C Leitão, António M Monteiro, Mikel Izquierdo, António J Silva, Estela Bastos, Mário C Marques

**Affiliations:** 1 Department of Sport Sciences, University of Trás-os-Montes and Alto Douro, Vila Real, Portugal; 2 Research Centre in Sports, Health and Human Development, Vila Real, Portugal; 3 Department of Health Sciences, Public University of Navarra, Campus of Tudela, Av. de Tarazona s/n., 31500 Tudela, Navarra, Spain; 4 Department of Sports Sciences, University of Beira Interior, Covilhã, Portugal; 5 Institute for Biotechnology and Bioengineering, Centre for Genomics and Biotechnology, University of Trás-os-Montes and Alto Douro, Vila Real, Portugal; 6 Department of Sport Sciences, Polytechnic Institute of Bragança, Bragança, Portugal; 7 CICS-UBI, Health Sciences Research Center, Covilhã, Portugal

**Keywords:** Resistance training, Angiotensin converting-enzyme, Alpha-Actinin-3, Women, Lower mobility

## Abstract

**Background:**

We studied the influence of the ACE I/D and ACTN3 R577X polymorphisms (single or combined) on lower-extremity function in older women in response to high-speed power training.

**Methods:**

One hundred and thirty-nine healthy older Caucasian women participated in this study (age: 65.5 ± 8.2 years, body mass: 67.0 ± 10.0 kg and height: 1.57 ± 0.06 m). Walking speed (S10) performance and functional capacity assessed by the “get-up and go” (GUG) mobility test were measured at baseline (T1) and after a consecutive 12-week period of high-speed power training (40-75% of one repetition maximum in arm and leg extensor exercises; 3 sets 4–12 reps, and two power exercises for upper and lower extremity). Genomic DNA was extracted from blood samples, and genotyping analyses were performed by PCR methods. Genotype distributions between groups were compared by Chi-Square test and the gains in physical performance were analyzed by two-way, repeated-measures ANOVA.

**Results:**

There were no significant differences between genotype groups in men or women for adjusted baseline phenotypes (P > 0.05). ACE I/D and ACTN3 polymorphisms showed a significant interaction genotype-training only in S10 (P = 0.012 and P = 0.044, respectively) and not in the GUG test (P = 0.311 and P = 0.477, respectively). Analyses of the combined effects between genotypes showed no other significant differences in all phenotypes (P < 0.05) at baseline. However, in response to high-speed power training, a significant interaction on walking speed (P = 0.048) was observed between the “power” (ACTN3 RR + RX & ACE DD) versus “non-power” muscularity-oriented genotypes (ACTN3 XX & ACE II + ID)].

**Conclusions:**

Thus, ACE I/D and ACTN3 R577X polymorphisms are likely candidates in the modulation of exercise-related gait speed phenotype in older women but not a significant influence in mobility traits.

## Background

The over-sixty year-old segment of the population exhibits an increased susceptibility to mobility limitations [1]. Aging also leads to a slowed capacity to develop high velocity movements and perform physical tasks to maintain independent functioning [2-4]. Two systematic reviews [1,5] reported that a slower walking speed is predictive of mobility limitations and a higher subsequent risk of fracture, institutionalization and mortality [6,7]. In older populations, functional capacity declines with advancing age, especially in older women [8], which increases the importance of rehabilitation and prevention programs to increase strength, mobility and balance confidence.

Like any other type of therapeutic intervention, exercise-training adaptations in older population are specific to the type of stimulus [9,10]. In the case of the lower limbs, previous studies have reported relevant increases in muscular power and functional capacity following resistance-training programs of variable durations in older women [8,11]. In addition, maximal strength and power in individual limbs are strongly correlated with mobility in older adults [12].

Improved knowledge of the specific mechanisms that mediate impairments in physical functioning, especially the relevance of functionality tests (gait speed versus mobility), based on demonstration of strength and muscle power, are crucial for developing effective interventions for preserving mobility and independence among older people [8,12].

Molecular analysis in older people can offer the opportunity to assess nonconventional exercise and lifestyle changes in an attempt to prevent muscular, metabolic or cardiovascular diseases. Several studies in different populations [13-18], [19,20] have confirmed that there is a considerable genetic component to the trainability of exercise performance and functional muscle properties. Indeed, multiple nuclear markers have been significantly associated and genetically determined as indicators of physical performance and muscular speed [10]. According to previous studies, both the angiotensin-converting enzyme (ACE) I/D and alpha-actinin 3 (ACTN3) R/X polymorphisms seem to be strong candidates to influence skeletal muscle phenotypes in older populations [16,18,21]. In fact, we have recently observed an effect of both ACE and ACTN3 genetic variants (individually or in combination) on muscle power gains and functional capacity in older Caucasian women in response to a high-speed power training [21]. Hence, the aim of the present study was to expand our previous results exploring a parameter of gait speed (walking 10 m distance, S10) and another mobility test (“go up and go” test, GUG) essential in maintaining balance and preventing falls in older populations. Both are components of predicted mobility limitations in the lower extremities and are fundamental in reflecting muscle function in older people [22].

To our knowledge, no association study exists that addresses genetic variation in mobility tests. Our current working hypothesis was focused on examining the influence of the ACE I/D and ACTN3 polymorphisms, alone and in combination, on walking speed and “get-up and go” test times in older Caucasian women in response to a consecutive 12-week period of high-speed power training stimuli. Thus, the purpose of this study was to determine the association of the combination of ACE I/D and ACTN3 R577X genotypes with gait speed and mobility trait in older women and to determine if the association of these polymorphisms may partly explain the interindividual variability in muscle function adaptation to resistance training.

## Methods

### Experimental design and approach to the problem

The goal of the present study was to expand our previous results comparing the influence of the ACE I/D and ACTN3 polymorphisms, alone and in combination, on walking speed 10 m distance (S10) performance and functional capacity as assessed by the “get-up and go” test, walking 2.44 m, turning, and returning to seated position (GUG) in older Caucasian women in response to a consecutive 12-week period of high-speed power training. A detailed description of the testing procedures has been given elsewhere [8]. To test the stability and reliability of the performance variables, a given section of the sample was consistently evaluated at the same time and location and supervised by the same researchers at pre- and post-intervention. Subjects were evaluated twice before the start of training (weeks -2 and 0), and this served as a control period (T1). In addition, we had previously tested the stability and reliability of these variables using a larger number of subjects over a control period in older women [8]. The same tests were then applied after the 12-week experimental period (T2).

### Subjects

One hundred thirty-nine healthy older Caucasian women participated in this study (age: 65.5 ± 8.2 years, body mass: 67.0 ± 10.0 kg and height: 1.57 ± 0.06 m). None of the participants had a history of strength training. The study subjects underwent a resistance-training program comprising three training sessions per week over 12 weeks based on the program developed by Pereira and colleagues [21]. Inclusion criteria for this group were: to be woman, older and no history of strength training. Before inclusion in the study, all candidates were thoroughly screened by a physician. Exclusion criteria included metallic prosthesis implants, artificial pacemakers, smoking habit, hip replacement surgery, walking only with assistance, and metabolic or endocrine disorders known to affect the musculoskeletal system. Each volunteer answered a face-to-face questionnaire addressing medical history, hormone replacement therapy, lifestyle habits, and medication use. All participants were of the same Caucasian ancestry over at least three generations. The experimental procedures were approved following the Helsinki Declaration and have been performed with the approval and Ethics Committee of Research Centre in Sports, Health and Human Development, of the University of Trás-os-Montes and Alto Douro, Department of Sport Sciences (reference number 002/2012). Written informed consent was obtained from each participant for the permission to use their information for the present report.

### Genotype assessment

During fall 2010, blood was collected in regular filter paper by finger blood spot (Albet, DP 400200), stored in separate plastic bags at 4°C until DNA extraction. Chelex 100® protocol (BioRad Laboratories, Hercules, CA) was used to extract DNA [23]. We followed the ACE I/D (rs1799752) and ACTN3 R577X (rs1815739) polymorphisms and were amplified by polymerase chain reaction (PCR), and the resulting PCR products were genotyped using agarose gel electrophoresis. The primers used for the ACE I/D polymorphism were F-5′-CTGGAGACCACTCCCATCCTTTCT-3′ and R-5′-GATGTGGCCATCACATTCGTCAGAT-3′. The ACE I/D fragments without insertion (Dallele) and with insertion (I-allele) of 190 and 490 bp, respectively, were detected on a 1.5% agarose gel containing ethidium bromide. For ACTN3 R577X polymorphism genotyping, a fragment of 291 bp was amplified with the following primers: ACTN3-F 5′-CTGTTGCCTGTGGTAAGTGGG-3′ and ACTN3-R 5′-TGGTCACAGTATGCAGGAGGG-3′. ACTN3 genotypes were established by enzymatic digestion of amplicons with Dde I. The R577X change creates a restriction site resulting in fragments of 108, 97, and 86 bp. Digestion of the R577 allele results in fragments of 205 and 86 bp, and digestion of the 577X allele results in fragments of 108, 97, and 86 bp. The fragments were detected on a 3% agarose gel containing ethidium bromide.

### Phenotype assessment

Tests were applied at two intervals: before the start of the 12-week experimental period (T1) and at its close (T2). Before testing, subjects were familiarized with all testing procedures, preceded by a general warm-up routine.

*Anthropometric measures:* total height (m) and body weight (kg) were assessed according to international standards for anthropometric assessment. To evaluate height a stadiometer (SECA, model 225, Germany) with a range scale of 0.10 cm was used and body mass (kg) was measured to the nearest 0.1 kg using a digital scale (Philips, type HF 351/00).

*Gait speed measure:* was measured with subjects instructed to perform three maximum effort sprints of 10 m (S10) beginning 2 m before the start line, in order to achieve optimal velocities over the test distance. Time at 0–10 m (S10) was recorded using Micrograte equipment (Racetime2 Light Radio Kit, USA). Subjects performed trial sprints, separated by 3 min of rest, on an indoor rubberized track.

*Mobility measure*: was evaluated over a walking distance of 2.44 m, characterized by “get-up and go” test (GUG). This test enables dynamic balance assessment, represented by the total time required for the subjects to rise from a seated position, walk eight feet (2.44 m), turn around and return to a seated position. The test was visually demonstrated in advance, and each subject was allowed to practice it once than two test trials were administered.

The test–retest reliability for S10 and GUG measurements taken was performed; the intra-class correlation coefficient (ICC) was always higher than 0.90.

### High-speed power training protocol

The resistance program (RT) consisted of progressive loads by 3 sets of 12 reps with a load of 40% of 1RM at the outset of a predetermined 1-repetition maximum up until 3 sets of 4 reps with load of 75% towards the end of the 12-week period in 1RM_LE_ and 1RM_BP_. For maximal strength, subjects also performed two explosive exercises for the upper and lower limbs [8]. All exercise training finished with abdominal crunches and trunk extensors. Sessions started and finished with general flexibility exercises. Rest intervals of 2 minutes between sets and 3 minutes between exercises were deployed. Verbal encouragement was given to ensure maximal contractions.

### Statistical analysis

Standard statistical methods were used for the calculation of means and standard deviations. Differences in the distributions of the ACE and ACTN3 genotypes were examined using Pearson’s x^2^. For Hardy-Weinberg equilibrium calculations, x^2^ statistic (one degree of freedom) was computed from the observed distribution of genotypes and the distribution of genotypes expected from applying the Hardy-Weinberg equilibrium assumption to the observed allele frequencies in the population. Parameters measured during exercise strength/power testing and functional performance achieved at the beginning and end of high-speed power training were analyzed by one-way ANOVA after checking for normality by Kolmogorov-Smirnov and for homogeneity of variance by Levene’s test. The two-way repeated-measures ANOVA, was used in each performance measure as the within-subjects variable and ACE and ACTN3 genotype as the between-subjects variable. Post hoc testing for significant differences in the ANOVA was performed by the Tukey’s honestly significant difference test. The combined effect of ACE I/D and ACTN3 R577X polymorphisms on the study phenotypes by ANOVA was analyzed using two genotype combinations, i.e. ACE DD and ACTN3 RR + RX (which hypothetically might be more suitable for power/hypertrophy-oriented exercise tasks) versus ACE II + ID and ACTN3 XX group [21]. Test-retest reliabilities, as shown by ICC, ranged from 0.90 to 0.93 for all testing exercises. Statistical significance was accepted at p ≤ 0.05 for all analysis. All data was analyzed using SPSS 17.0.

## Results

The Hardy-Weinberg equilibrium (P = 0.125 and P = 0.06, respectively) and the distribution of allelic frequencies for ACE I/D and ACTN3 R577X genotypes in our cohort study were 0.56 and 0.44 for the D- and I-alleles, respectively, and 0.60 and 0.43 for the R- and X-alleles, respectively. At baseline (T1) and after the resistance training (T2), no significant differences (P > 0.05) in either ACE or ACTN3 genotypes were observed for anthropometric measures [21,24].

Table 1 shows the ACE genotype influence on performance gains. From pre- to post-training period, subjects increased their muscle power output by maximal velocity in S10 (ID: 10.9%; DD: 14.3% and II: 10.2%), as well in functional capacity measured by means of the GUG test (ID: 13.2%; DD: 9.6% and II: 7.5%). Indeed, significant training effects (within subject) were noted for both walking speed and the GUG test (P < 0.05). Moreover, despite the significant differences among genotypes for both tests in T2 the effect of genotype on performance gains were only significant for S10 (P = 0.012) and not for the GUG test (P = 0.331).

**Table 1:** Effect of ACE genotype in older Caucasian women in test performance gains during training

		**Moments**		**Repeated measures 2-way ANOVA**	
**Performance measures**	**ACE genotype (n = 139)**	**T1**	**T2**	**Training effect (within subjects) P values**	**Genotype effect (between subjects) P values**
		**х±σ**	**х±σ**		
**S10 (m/s)**	**ID (n = 52)**	5.5 **±** 0.7	4.9 **±** 0.6^a^	0.001	0.012
	**DD(n = 52)**	5.6 **±** 0.7	4.8 **±** 0.6^b^		
	**II (n = 35)**	5.9 **±** 0.8	5.3 **±** 0.8		
P*		0.060	0.006		
**GUG (sec.)**	**ID**	5.3 **±** 0.9	4.6 **±** 0.5	0.001	0.311
	**DD**	5.2 **±** 0.8	4.7 **±** 0.7		
	**II**	5.3 **±** 0.8	4.9 **±** 0.7		
P*		0.721	0.013		

Legend: Values are mean ± SD; *n*, no. of subjects. Pre-training and post-training, before (T1) and after (T2) 12-week of high-speed power training, respectively. All reported values were measured at maximum effort. *P values (P < 0.001) were computed by one-way and 2-way ANOVA. ^a^P < 0.001, ID significantly different from II; ^b^P < 0.001, DD significantly different from II.

Similar results are shown in Table 2, respecting ACTN3. From pre- to post-training, subjects significantly increased their muscle power output by maximal velocity in S10 (RX: 10.9%; RR: 14.3% and XX: 11.9%), as well as in functional performance (RX: 11.3%; RR: 11.5% and XX: 7.5%). Significant group × time interactions (P < 0.05) were observed for ACTN3 R577X. Significant training × genotype effects of the ACTN3 R577X polymorphism were also observed in S10 (P = 0.044) but not for the GUG test (P = 0.477).

**Table 2:** Effect of ACTN3 genotype in older Caucasian women in test performance gains during training

		**Moments**		**Repeated measures 2-way ANOVA**	
**Performance measures**	**ACTN3 genotype (n = 139)**	**T1**	**T2**	**Training effect (within subjects) P value**	**Genotype effect (between subjects) P value**
		**х±σ**	**х±σ**		
**S10 (m/s)**	RX (n = 54)	5.5 **±** 0.7	4.9 **±** 0.6^a^	0.001	0.044
	RR (n = 52)	5.6 **±** 0.6	4.8 **±** 0.6^b^		
	XX (n = 33)	5.9 **±** 0.9	5.2 **±** 0.9		
P*		0.080	0.050		
**GUG (sec.)**	RX	5.3 **±** 0.9	4.7 **±** 0.7	0.001	0.477
	RR	5.2 **±** 0.8	4.6 **±** 0.5		
	XX	5.3 **±** 0.8	4.9 **±** 0.7		
P*		0.615	0.139		

Legend: Values are mean ± SD; *n*, no. of subjects. Pre-training and post-training, before (T1) and after (T2) 12-week of high-speed power training, respectively. All reported values were measured at maximum effort. *P values (P < 0.001) were computed by one-way and 2-way ANOVA. ^a^P < 0.001, RX significantly different from XX; ^b^P < 0.001, RR significantly different from XX.

**Figure 1:**
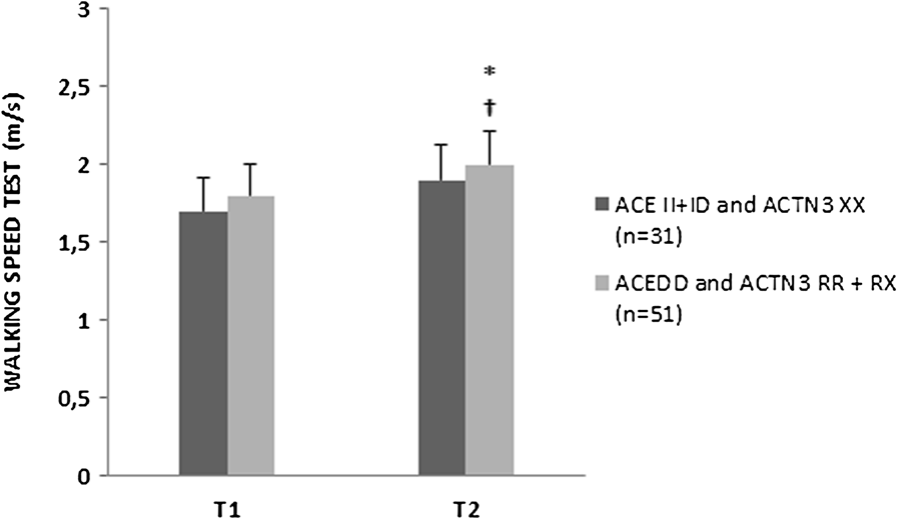
**Walking velocity test at the beginning of the protocol (T1) and after 12-weeks (T2).** Data presented are mean ± SD. *Significantly different (P ≤ 0.05) between T1 and T2 weeks; †Significant changes (P ≤ 0.05) between the groups.

**Figure 2:**
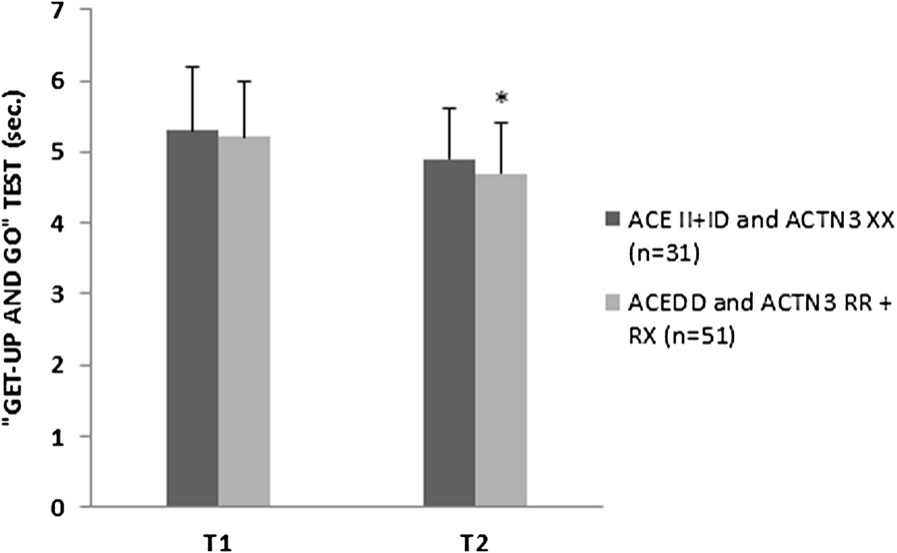
**“Get-up and go” test at the beginning of the protocol (T1) and after 12-weeks (T2).** Data presented are mean ± SD. *Significantly different (P ≤ 0.05) between T1 and T2 weeks; †Significant changes (P ≤ 0.05) between the groups.

The analyses of the combined effects between genotypes ACTN3 RR + RX & ACE DD versus ACTN3 XX & ACE II + ID did not show a significant difference (P > 0.05) at baseline. However, after 12-weeks of high-speed power training, a significant influence on walking speed (P = 0.048) though not in the GUG test (P = 0.318) (Figures 1 and 2) were observed in combined ACE and ACTN3 genotypes.

## Discussion

This study was the first to examine the influence of the ACE and ACTN3 genotypes on training-induced changes in gait speed and mobility before and after a high-speed power training period in older Caucasian women. A distinctive finding of the present study was the evidence that ACE I/D and ACTN3 R577X polymorphisms, individually or in combination, have a significant influence on gait speed phenotype in older Caucasian women but not a significant influence in mobility trait in response to high-speed power training.

The large variability in training-induced adaptations suggests that molecular mechanisms also govern the major part of muscle variation in exercise [10,22,25]. Insights concerning the ACE I/D and ACTN3 R577X polymorphisms’ influence on the modulation of exercise-related phenotypes in adults are controversial. In the past decade, some studies have shown a positive effect [13,26] on older subjects, particularly in response to resistance training. However, several other studies have failed to support a positive association [25,27,28]. Our study, besides contributing to the clarification of this discrepancy in the literature, focused on the effects of ACE and ACTN3 genotypes on training-induced changes in lower-extremity function in older Caucasian women. The present study also stated that according to lower extremity performance is a common weakness in older people [4] and our molecular analysis can improve knowledge of the specific physiological mechanisms that mediate impairments in physical functioning. Present data shows that high-speed power training is an effective therapeutic intervention. ACE I/D and ACTN3 R577X polymorphisms seem to influence variations in walking velocity, an essential factor in retaining mobility and maintaining reaction time in power tasks such as crossing the road or avoiding a fall. However, the results of our study failed to support any significant genetic influence for either polymorphism (single or combined) in balance and mobility skills as measured by GUG test performance.

At baseline (T1) no differences were observed in S10 and the GUG test. Nevertheless, at post-intervention (T2), significant differences between ACE I/D genotypes were confirmed.

There was a significant interaction between ACE genotype and S10 after 12-weeks of high-speed power training in older women, with greater improvements in velocity shown by subjects with the D allele (DD and ID genotypes). However, as ACE II genotype has a lower number of subjects, this might play some role in the results.

As previously reported by Gordon et al. [29], ACE catalyses the production of angiotensin II which mediates the hypertrophic response via the AT-1 receptor. As research over the past decade has shown, the ACE gene is associated with optimal muscle function, particularly in strength at high velocity [30] such as sprint time performance [23,31-33]. According to this, our data showed that individuals with ACE*ID and ACE*DD genotypes presented values suggesting superiority in the power velocity test (S10) compared to those with the ACE*II genotype, both before and after the training program. However, owing to complex effects, ACE I/D genotype-training interaction did not influence mobility capacity as measured by the GUG test. On performing this experiment, older women tend to focus on maintaining balance in rising from the chair and may disregard high velocity execution. Also might be that the posture control (i.e. sensorial system) play a major role in it. Nevertheless, the changes that occur in skeletal muscle structure and function with aging may partial explain for the lack of a significant relationship between mobility phenotypes and the ACE I/D polymorphisms.

Concerning to the ACTN3 R577X polymorphism, a significant gene*training interaction was also evident only in S10 and not in the GUG test. Other studies have reported similar results regarding the association between the ACTN3 genotype and sprint performance in populations of varied ethnicity, including European, American, and Israeli athletes [14,34-36]. Because ACTN3 expression is limited to type II fibers, the measurement of S10 might be the best phenotype for testing the influence of the ACTN3 polymorphism over typical mobility measures. According to the physiological role of ACTN3 in speed and contraction velocity, we hypothesized those participants who were ACTN3 deficient (XX) would not be as strong or produce as much instantaneous power responses to high-speed power training as would R-allele carriers. At baseline, the women homozygous for the ACTN3 mutant allele 577X (XX) showed lower velocity in S10 and GUG tests compared to the other genotypes. After 12-weeks of high-speed power training, women homozygous for the wild type (RR) and heterozygous (RX) demonstrated superior performance in both measures compared with the homozygous XX. Indeed, velocity were greatest for RR and least for RX or XX polymorphisms, in the R577X genotype was observed in the present study. As in previous reports [13,15,17,25] our results confirm that alpha-actinin-3 deficiency appears to impair muscle performance. Also, the lack of alpha-actinin-3 may indicate faster decline in muscle function with increasing age [19] and selective atrophy of type II muscle fibers in advanced age could attenuate any influence of the ACTN3 genotype on muscle function [26]. Moreover, as Clarkson et al. [26] suggested, our study confirms that subjects lacking in ACTN3 do not increase power as do those having the 577R allele.

Norman et al. [37], as previously stated that repeated exercise may show an increase in power performance in RR, though not in XX genotypes, suggesting that the ACTN3 genotype may modulate responsiveness to training in women [38,39]. It seems that α-actinin-3 expression may affect muscular capacity, which implies that α-actinin-2 may compensate for this deficiency and neutralize the phenotypic consequences at baseline [13,25,39]. Indeed, our intervention program based on high-speed actions was essential in revealing a significant difference between ACTN3 genotypes on performance and response to training particular in older women. It seems that only precise testing (i.e. maximal strength, power) can demonstrate significant difference between subjects, specifically after a program training intervention.

Indeed, in older Caucasian women, the present data showed that ACE II + ID & ACTN3 XX versus ACE DD & ACTN3 RR + RX revealed significant differences (P < 0.05) only in an S10 test evaluated after 12-weeks of high-speed power training. A mean improvement of gait speed in subjects with D and R alleles may correspond with meaningful reductions in risk for these adverse outcomes. Concerning the GUG test, it would appear that this is a consistent measure for evaluating the prognosis of falls [40] but is not sufficiently sensitive to absorb a significant genotype-training interaction of both ACE I/D and ACTN3 R577X polymorphisms (alone or in combination). Balance and coordination skills are too complex to enable the isolation of a restricted polymorphic influence. Future studies are also necessary with larger samples of older males and young people in order to replicate the findings we obtained with our studies.

The present study has some limitations. First, our participants were limited to older women, and do not permit to study the influence of gender in genetic association with muscular phenotypes. Secondly, according to muscle function, these measures should be analyzed in older people with functional limitations to observe if the training has the same effect in the present genotypes.

## Conclusion

In summary, this study has shown that both ACE I/D and ACTN3 R577X polymorphisms (alone or in combination) are strong candidates in the modulation of power phenotypes such as walking velocity in response to high-speed power training in older Caucasian women. In functional measures such as the “get-up and go” test that does not focus on a restricted power contraction, significant differences between genotypes are not easily identified. Our study confirms the importance of analyzing the effects of a 12 week intervention in gait speed and mobility in older people.

## Competing interests

The authors declare that they have no competing interests.

## Authors’ contributions

AP participated in designing the study, collecting and analyzing all data and drafting the manuscript. EB develop all genotyping methods and JCL revised statistic data. MCM and AJS participated in designing the study and treatment protocols and coordinate the study. AMC and MI revised the manuscript critically. Both authors read and approved the final manuscript. AMM coordinates the recruitment of participants. All authors read and approved the manuscript.

## Pre-publication history

The pre-publication history for this paper can be accessed here:

http://www.biomedcentral.com/1471-2318/13/131/prepub
